# Computed tomography bone density variations in oncological patients undergoing antiresorptive medication

**DOI:** 10.4317/medoral.25191

**Published:** 2022-10-16

**Authors:** Wellington Hideaki Yanaguizawa, Francisco Barbara Abreu Barros, Solange Kobayashi-Velasco, Fernanda Cristina Sales Salineiro, Fábio Abreu Alves, Marcelo Gusmão Paraiso Cavalcanti

**Affiliations:** 1 Department of Stomatology, School of Dentistry, University of São Paulo, São Paulo, SP, Brazil; 2 A. C. Camargo Cancer Center, Stomatology Department, São Paulo, SP, Brazil

## Abstract

**Background:**

The purpose of this study was to compare jaw and cervical vertebrae bone density in computed tomography (CT) analyses of oncological patients undergoing antiresorptive medication with control patients, aiming to find information that may assist the radiologist and clinician in predicting risks and monitoring osteonecrosis in the jaw.

**Material and Methods:**

Thirty-one patients treated with zoledronic acid and 37 control were included in the study. Two areas in regions of interest were chosen and standardized, one in the lower portion of the mandible and another in the axial cervical vertebra (C2) of patients undergoing antiresorptive drug treatment (experimental group) and the control group. Density analysis was performed using Hounsfield scale grayscale values obtained from multislice CT exams. Interclass correlation coefficient test (ICC) was performed to assess reproducibility and repeatability. The test of normality of the samples was demonstrated using the Shapiro-Wilk test and the comparison performed using Mann-Whitney U non-parametric test.

**Results:**

When compared to patients in the control group, patients undergoing antiresorptive medication depicted an increase in bone density in both jaw bone (*p*=0.021) and cervical vertebrae (*p*=0.002). The same pattern could be observed in patients who used the medication on a monthly basis for analysis of jaw bone (*p*=0.021), the cervical vertebrae (*p*=0.002), and the cervical vertebrae of the patients who used the medication on a quarterly basis (*p*=0.003).

**Conclusions:**

CT can be a potentially useful method for detecting alterations associated with antiresorptive therapy, serving as a possible tool in the prediction of the disease progression.

** Key words:**Bone density, computed tomography, bisphosphonate osteonecrosis, jaw, cervical vertebrae.

## Introduction

Antiresorptive drugs are widely employed in the treatment of osteoporosis and bone metastasis. However, they may cause severe side effects, such as medication-related osteonecrosis of the jaw (MRONJ) (1). The diagnosis of osteonecrosis is based on the association of clinical, histological and radiographic investigations. Clinically, MRONJ is defined as exposed necrotic bone or active fistulae in the maxillofacial area that persists for more than 8 weeks; other signs include local edema, purulent secretion and painful symptoms, with the jaw bones being the most affected sites (2).

Imaging features of MRONJ include: areas of osteolysis, sclerosis or a mix of both, disorganization of the medullary trabecular bone and/or changes in the cortex. In advanced cases, it may present areas of bone sequestration, periosteal reaction and pathological fracture (3). The quality of life of patients with MRONJ is significantly affected and its treatment is not always simple, especially in later stages. For instance, surgical treatment has a high success rate, getting superior results when compared to drug treatments or more conservative treatments such as the application of topical chlorhexidine, low-power laser therapy sessions and hyperbaric camera. Therefore, disease prevention and correct planning in dental care are crucial for patients undergoing therapy with bisphosphonates (4).

Up to the present study, there are no complementary tests reliable enough to predict the risk rates of bone necrosis development. The C-terminal telopeptide (CTX) exam, which consists of a serum marker for bone resorption, was previously used as a parameter prior to surgical-invasive dental care for these patients. However, systematic reviews have revealed that this is an unreliable exam in predicting MRONJ risks (5).

In contrast, some studies carried out with imaging exams, mainly computed tomography (CT), suggested that these exams may be an interesting tool for the assessment of variations in bone density influenced by medications, thus serving as a possible marker in the prediction of possible illnesses (6-8). Jaw bone, however, seems to have limitations in density assessment due to osteonecrosis interferences. Cervical vertebra bone could be an interesting place for this analysis. However, in the literature review, no studies concerning cervical vertebra bone density analysis in patients under antiresorptive medication have been published.

The purpose of this study was to compare jaw and cervical vertebra bone density on CT analysis of oncological patients undergoing antiresorptive medication with control patients. In this comparison, we aimed to find information that might assist the radiologist and clinician in predicting risks and monitoring MRONJ cases.

## Material and Methods

This is a retrospective study that assessed CT follow-up exams requested by the oncologist: 31 patients with prostate, breast, kidney, or liver cancer under zoledronic acid 4 mg intravenously therapy in various timeframes (test group), and 37 patients with tongue or oropharynx cancer, who had never used antiresorptive or antiangiogenic drugs (control group).

All patients were classified according to the MRONJ stage guidelines provided by the American Association of Oral and Maxillofacial Surgeons (AAOMS) (1). Patients classified as stage 3, with extensive osteolysis and pathological fractures were excluded from the study. Also, patients who had undergone radiotherapy treatment in the head and neck region, with evidence of remaining tumor or tumor recurrence in the region of jaw or cervical vertebrae, with osteonecrosis lesions close to the portions of the base of the jaw, or patients who had presented any condition that might have influenced their systemic bone density (osteopenia, osteoporosis, multiple myeloma, etc.) were excluded from this study. The patients were referred for CT exams in a multislice computed tomography (MSCT) device (Brilliance 64; Philips Healthcare, Cleveland, OH, USA) located in the Radiology Department of our Institution, from 2016 to 2019, at 120 kVp and 200 mA, 512 x 512 matrix, using bone filter. The reconstructed interval was 1.0 mm yielding overlapping, with 2mm slice thickness. Data sets (DICOM – Digital Imaging and Communications in Medicine format) were transferred, to an independent workstation, (OsiriX MD software, version 10.0.1 for MacOS, Pixmeo Sarl, Geneva, Switzerland) to generate multiplanar reconstructed (MPR) images simultaneously, in order to perform the imaging analysis.

**Figure 1:**
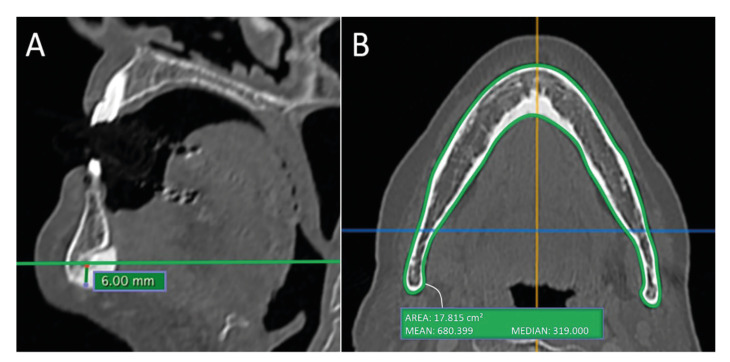
A) Computed tomography (CT) measurement method, 6 mm above the base of the mandible (sagittal image). B) Region of interest (ROI) was set on the mandibular body regions contour including medullary and buccolingual cortical bones - mean and median CT grayscale values (axial image).

Density analysis was based on Hamada *et al*. (7) and Taniguchi *et al*. (8) studies. A particular axial slice that did not present bone necrosis images was selected and standardized as region of interest (ROI), both for mandible and for axial cervical vertebra (C2). To assess the ROI in the mandible region, 3 points at the base of the mandible were determined: two symmetrical points at the base of the mandible, located at the lowermost prominent area of the mandible angle, and a single point in the region of the mandibular symphysis in the sagittal and axial images was also projected to the base of the mandible. The plane formed by the three points was aligned parallel to the horizontal plane. Then, in the sagittal image, 6 mm was measured vertically upwards from the outer edge of the cortical at the base of the mandible. With the axial image at the height of 6 mm, the outline of the entire mandible was delimited in the axial image, generating a HU value (Fig. 1).

**Figure 2:**
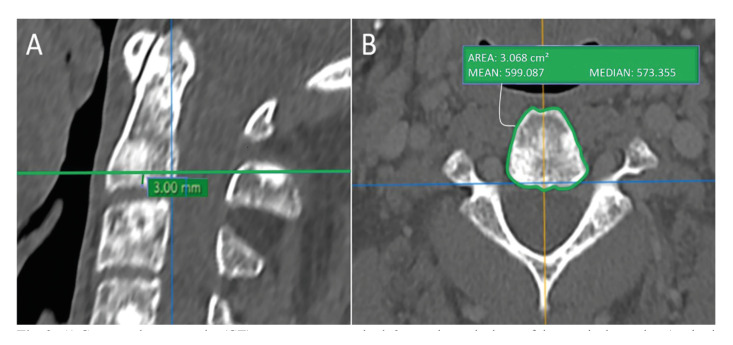
A) Computed tomography (CT) measurement method, 3 mm above the base of the cervical vertebra (sagittal image). B) Region of interest (ROI) was set on the C2 body region contour including medullary and cortical bones - mean and mean CT grayscale values (axial image).

To determine the ROI in the region of the cervical vertebra, 4 points at the base of C2 were established, with the vertebra positioned in the midline by the axial plane. The horizontal plane was aligned at the lowest points of the base of the body of the vertebra through the coronal and sagittal slices. Consequently, the plane formed by the four points should be parallel to the horizontal plane. In the sagittal section, 3 mm was measured vertically upwards from the outer edge of the cortical at the base of C2. With the axial cut at the height of 3 mm, the entire body of the cervical vertebra was delimited thus generating a HU value (Fig. 2).

Values obtained by the ROI tool at both test and control groups were compared. Due to the physiological bone changes over time and biological differences between male and female patients, test and control groups were matched by sex and age. Two professionals (an oral and maxillofacial radiologist and an oral medicine specialist) evaluated each volume, independently. They had been previously calibrated according to the methodological parameters used for the analysis of bone density of the mandible and vertebra. After two weeks, the two examiners repeated the same assessment (intra-examiner evaluation).

Interclass correlation coefficient test (ICC) was performed to assess reproducibility and repeatability. The analyses were performed using the average between both values obtained by each observer at different times. Shapiro-Wilk test was used to assess the normality of the samples. Based on its results, the comparison between test group and control group in both mandible and vertebra were performed using the Mann-Whitney U non-parametric test. Values of *p* <0.05 were considered as indicative of statistical significance.

## Results

The patients’ demographics are summarized in Table 1. The most common diseases were prostate [10] and breast cancer [19]. Among patients submitted to bisphosphonate drug therapy, 74.2% presented images suggesting MRONJ. Tooth extractions were the most frequent event associated with the development of MRONJ, followed by trauma caused mainly by prostheses. Other factors such as implant installation and spontaneous origin have also been reported. Among the patients included in this study, 61.2% that had not previously had dental evaluation before cancer treatment and 74.2% that had undergone bisphosphonate drug therapy, ended up developing MRONJ. Regarding the staging of MRONJ, 25.8% of the cases were classified as risk and / or stage 0, 29.1% in stage 1 and 45.1% in stage 2.

**Figure 3:**
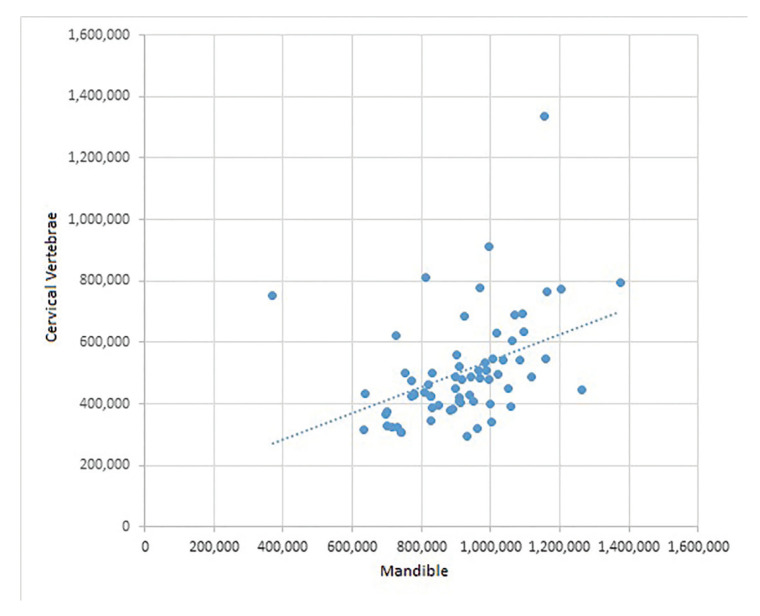
Distribution of the mean bone density values of the mandible and vertebra.

Observers were considered excellent in both inter- and intra-rater analyses, based on interclass correlation coefficient test (reference value > 0.75). The mean values of HU scale found in the mandible and vertebra were distributed in a directly proportional way. Thus, high density values in the jaw bone correspond to high density values in the vertebra bone. Vertebra grayscale values had less variation in its distribution when compared with mandible (Fig. 3).

In the comparative analysis between bone densities, higher HU scale values were found in the group that had received antiresorptive medication compared to the control group, and these differences were both statistically significant for the analysis of bone density in the mandible (*p*=0.021) and for the cervical vertebrae (*p*=0.002). The same pattern was observed when we compared the patients who used the medication on a monthly basis for density analysis of jaw bone (*p*=0.021), the cervical vertebrae (*p*=0.002), and the cervical vertebrae analysis of the patients who used the medication on a quarterly basis (*p*=0.003). There was no statistically significant difference between control group and analysis of jaw bone that patients who had received medication on a quarterly basis (*p*=0.079) (Table 2).

**Table 1:**
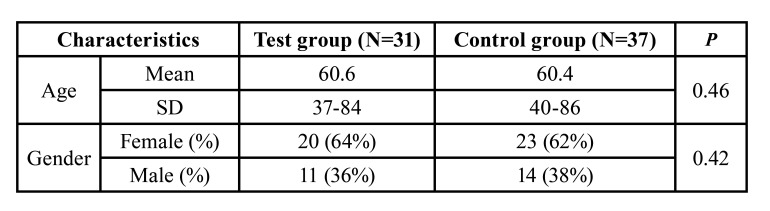
MRONJ cases characteristics.

**Table 2:**
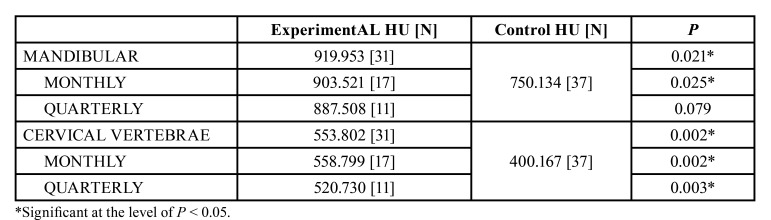
Bone density in Hounsfield Units comparison in both mandible and cervical vertebra.

## Discussion

MRONJ pathophysiology remains unknown, but many authors support the hypothesis that the decrease in vascularization and changes in bone tissue remodeling caused by the use of antiresorptive and antiangiogenic drugs may be related to the favoring of conditions for the development of bone necrosis lesions (9-11).

Antiresorptive drugs, such as bisphosphonates, have beneficial therapeutic effects in the treatment of hypercalcemia induced by malignant tumors or in the control of bone loss resulting from metastatic skeletal lesions. In these situations, bisphosphonates are often prescribed in injecTable administration, due to the greater need for concentration and potency of the medication (12). All patients included in this study had a diagnosis of malignant tumor, so they were all treated with zoledronic acid 4 mg intravenously.

The good condition of oral hygiene in patients that have used bisphosphonates, especially in injecTable doses, is essential to prevent MRONJ. Therefore, studies are unanimous in recommending dental evaluations before and during the use of medication, performing prophylaxis, periodontal scaling, root canal treatment and extractions, eliminating possible foci of inflammation and oral infection (13,14). In the present study, 61% of patients who developed osteonecrosis lesions reported not having undergone dental evaluation before starting medication.

Two studies analyzed histological sections and Micro CT images and demonstrated a positive association between the application of injecTable zoledronic acid and increased bone density in rats. The researchers observed smaller spaces in the trabeculae of the medullary jaw bone, which may have generated a structure with less vascularization compared to the control group, which did not use the medication (6,15-19). However, another group of researchers did not find this difference in grayscale values when using cone beam computed tomography (CBCT) to assess the mandible and tibial density of rats subjected to injecTable zoledronic acid (20).

CBCT is frequently employed to assess bone volume and density in cases of implant planning and evaluation during dental rehabilitation, however studies show that this imaging modality is not valid for measuring bone density only (21). In addition, a study observed that the grayscale values generated by CBCT seem to be unreliable, since they were much higher than the values obtained through the Hounsfield Unit scale (HU) in MSCT (22).

The analysis of bone density using HU scale obtained from the MSCT examination demonstrated correlated results similar to the scores found in the examination of bone densitometry by double emission of X-ray in patients with osteoporosis, considered the reference standard examination for investigation of bone density. Therefore, HU scale appears to be a reliable test for analyzing bone density (23). Based on this assertion, HU values were used to measure bone mineral density in this study.

Heim *et al*. (24) quantified the bone density at the specific scale of the mandible in axial section using the HU scale, one in the region close to the mental foramen and the other in the retromolar region. The results pointed to variations in bone microarchitecture in patients that were submitted to treatment with bisphosphonates compared to control patients. In addition, increase in bone density was greater in the group that had used bisphosphonates than in the group that had used denosumab.

Goler-Bulut *et al*. (25) compared the diameter of foramina and neurovascular channels of maxillary bones (such as the nasopalatine canal, mandibular canal and mental foramen) of patients who had undergone the use of bisphosphonates. They found a decrease in the diameter of the structures when compared with control patients through analysis with CBCT exam.

Mandible cortical bone also appears to develop imaging alterations with the use of antiresorptive medication. Torres *et al*. (26) demonstrated a significant increase in thickening at the cortical portion of the base of the mandible through CBCT exams in patients with MRONJ when compared with control patients.

Koo and Lee (27), using MSCT, also found an increase in the thickness of the cortical bone, from the average of the values found in the cortical of the base and the buccal and lingual walls of the mandible. The average thickness found in patients who had used bisphosphonates and developed MRONJ was 3.81mm, a statistically significant difference compared to the average of 3.23mm in cortical thickness found in patients in the control group. In our study, the axial plane parallel to the horizontal plane with a distance of 6 mm above the base of the mandible was used, thus preventing erroneous variations related to the thickness of the cortical of the mandibular base.

Likewise, other studies have observed these changes in bone density also in the medullary portion of the jaw bone, finding higher values of bone density, even in patients in early stages of the disease, using different methods of analysis and different (ROI) in the mandible from MSCT exams (7,8). In our analyses, as in other studies (8,21), we considered the total values of the cortical region added to the density values of the medullary bone region, in which we also found statistically significant differences between the bone densities of the mandible bone.

In contrast, Jain *et al*. (21) prospectively evaluated, using HU scale in CT scans of 57 patients who had received 6 monthly doses of zoledronic acid (4 mg) and compared the bone density present in 24 ROI of parasagittal slices located in the mandible and maxilla in 3 moments: before application, 6 months and 12 months after medication. Their results revealed a small increase in all assessments, but there were no statistically significant differences between mean bone densities in the cuts analyzed over the period of one year. However, a significant increase in bone density was observed between 6 months and 1 year in medullary bone in the anterior region of the mandible. In our study, we did not use parasagittal cuts because they presented a high degree of difficulty in the reproducibility and repeatability of ROIs.

The studies carried out by Hamada *et al*. (7) and Taniguchi *et al*. (8) found no statistical differences in bone densities between patients who had used the medication orally and patients who had received medication intravenously. The same studies also found no differences in the density of the cortical and medullary bone of the jaw among patients at different times of medication use, noting similar results both in individuals who used zoledronic acid for less than 3 years and for periods of 3 years or more (7,8). Due to the limited number of research participants in our study, patient analyses were not performed at different times and frequencies of medication use.

Some studies have evaluated other bone regions in patients who had used injecTable antiresorptives and found results similar to the studies carried out in maxillary and mandibular bones, observing an increase in density in these regions as well. Bransford *et al*. (28) performed density analysis in L6 and L7 lumbar vertebrae of rats using the HU scale on CT, revealing an increase in the bone mineral content of these regions. Quattrocchi *et al*. (29) evaluated HU values in axial sections of CT scans, both of the iliac bone and the sacrum bone, in patients submitted to injecTable zoledronic acid 4 mg for the treatment of bone metastases of malignant prostate tumors, finding an increase in bone density in these regions presumably generated by medication. The previously mentioned studies (28,29), corroborate our findings in terms of increase in bone density in both mandible and cervical vertebrae. Since C2 will always be present in CT scans in the head and neck region, and is not as susceptible to the initiating factors of MRONJ, it seems to be an interesting region to be assessed, as it is not influenced by large lesions, and has fewer anatomical and therapeutic interferences to perform analysis of bone mineral density of patients using antiresorptive drugs. This analysis may be performed in order to create a parameter of patients’ predisposition to develop or not MRONJ lesions in the oral cavity in the future. In addition, in our study, the vertebra analysis presented statistically significant increased values in different frequencies of medication use, whereas the mandible did not show the same result when comparing patients who used the medication on a quarterly basis.

Another interesting finding in our study was that patients classified as “at risk”, that is, who had used the medication, but did not develop MRONJ lesions, obtained density values equal to those of patients in more advanced stages of the disease. These patients were not subjected to the causative factors initiating MRONJ, but they might be at risk of developing the lesion due to changes in their bone mineral density compared to the control group. These interesting finding shows the importance of preventive care and also the maintenance of oral health during treatment, in order to avoid more invasive and complex procedures that may develop MRONJ lesions.

This research was a retrospective analysis and was restricted to the data provided in the hospital's electronic medical records, thus comprising a limitation of study. In addition, as it is a cross-sectional study, only data from a static situation of the bone condition were obtained, not allowing a comparison between the same patient, before and after the administration of antiresorptive medication. Additionally, there was a difficulty in including patients in the research due to the exclusion criteria of the study, since it is a disease caused by an adverse effect of the antiresorptive medication. Therefore, the statistical analysis did not present subgroups with significant numbers to add more variables, such as the stage of the disease and duration of medication use.

## Conclusions

CT exams of jaw bone and axial cervical vertebra bone density of oncological patients undergoing antiresorptive medication indicated grayscale alterations. Thus, the analysis by HU through CT examination seems to be a potentially useful method for detecting early changes associated with bisphosphonate therapy and for predicting cases in which necrosis may occur after a more invasive dental intervention. However, further research is needed to achieve more robust evidence.
